# Sulfate Freeze–Thaw Resistance of Magnesium Potassium Phosphate Cement Mortar

**DOI:** 10.3390/ma15093342

**Published:** 2022-05-06

**Authors:** Bin Yang, Rong-Jian Ji, Qian Lan, Jian-Ming Yang, Jun Xu

**Affiliations:** 1 School of Architecture Engineering, Jiangsu Open University, Nanjing 210036, China; yb86221120@163.com; 2 Yangzhou Polytechnic Institute, College of Architecture Engineering, Yangzhou 225127, China; lanq1020@163.com; 3 School of Civil Engineering, San Jiang University, Nanjing 210012, China; yjm_kk@163.com; 4 Department of Architecture and Civil Engineering, College of Civil Engineering and Architecture, Jiangsu University of Science and Technology, Zhenjiang 212000, China; xujun@just.edu.cn

**Keywords:** magnesium potassium phosphate cement mortar, sulfate freeze–thaw resistance, strength, weight loss, water absorption

## Abstract

Concrete facilities in the severe-cold areas of western China (salt lake environments and heavy saline soils) are seriously damaged by the multiple corrosion effects of freeze–thaw cycles and sulfate corrosion. Magnesium phosphate cement (MPC) cement-based material has become an ideal concrete structural component because of its superior performance. Because concrete structural repair materials are used in heavy-corrosion environments, their durability in those environments should also be considered. Regarding the salt-freezing resistance of MPC, the existing studies have all used a NaCl solution as the heat transfer medium. In addition to chlorine salt, sulfate, especially Na_2_SO_4_, is also common in typical use environments such as oceans, salt lakes, and groundwater. To evaluate the sulfate freeze–thaw resistance of potassium magnesium phosphate cement (MKPC) mortar, in this study the strength development, weight loss, and water absorption of MKPC mortar specimens subjected to different freeze–thaw cycles were tested and compared with those for Portland cement (P.O) mortar specimens of the same strength grade. The results showed that the P.O mortar specimen completely lost its strength after 75 cycles of rapid water freezing and thawing and 50 cycles of sodium sulfate solution (5%) freezing and thawing. However, the residual strength rating of the MKPC mortar specimen after 75 cycles of water freezing and thawing and 100 cycles of sodium sulfate solution freezing and thawing was higher than 75%. After 50 rapid freeze–thaw cycles in water and a 5% Na_2_SO_4_ solution, the P.O mortar specimen’s mass loss exceeded the 5% failure standard, whereas the mass loss of the MKPC mortar specimens was much less than 5%. Before the freeze–thaw cycles, the water absorption of the P.O mortar specimen was close to 8 times that of the MKPC mortar specimen, and after 50 water freeze–thaw cycles and 25 sulfate solution freeze–thaw cycles, the water absorption reached 4.88% and 5.68%, respectively. However, after 225 freeze–thaw cycles in water and the sulfate solution, the water absorption rates of MKPC mortar specimens were 2.91% and 2.51% respectively. The test and analysis results show that the freeze–thaw resistance of MKPC mortar was much higher than that of Portland cement mortar specimens. Those results provide a prerequisite for applying and expanding the use of MKPC-based materials in severe-cold areas of western China (salt lake and heavily saline soil environments).

## 1. Introduction

The main causes of concrete durability damage are reinforcement corrosion, freeze–thaw damage in cold areas, and a corrosive environment’s physical and chemical effects [1]. If water seeps through a breach into a hardened body of concrete so that it reaches a certain saturation state, and if the ambient temperature continues to alternate between freezing and above-freezing temperatures, the state of capillary pore water in the concrete changes at a certain below-freezing temperature, and the capillary pore wall is affected by frost heaving pressure (water freezing) and osmotic pressure (supercooled water migration). Those produce tensile stress in the microstructure around the breach, which eventually leads to deterioration in the concrete’s performance. Concrete freeze–thaw damage often occurs in various marine and hydraulic structures in cold areas, as well as in bridges and pavement that are often exposed to rainwater. That also seriously affects the normal service performance and long-term safety of buildings.

Similarly, deterioration in concrete performance caused by salt erosion is also quite serious and threatens the safety of many concrete structures in western China’s coastal and offshore areas. Among the several types of salt attacks on concrete, sulfate attacks are the most common. When environmental water infiltrates the concrete, SO_4_^2−^ reacts with its Ca(OH)_2_ and calcium aluminate hydrate to produce insoluble salts. Those salts absorb many water molecules and expand in volume. When the internal expansion stress exceeds the tensile strength of the concrete, the concrete is eventually destroyed. Sulfate erosion is extremely harmful to the concrete material itself, and the double action of freezing and thawing complicates the failure mechanism of concrete structures [1,2]. Portland cement-based material paste contains much Ca(OH)_2_, and the main hydration product, calcium-silicate-hydrate (C-S-H) gel, is porous. These inherent defects make Portland cement concrete no longer suitable for environments with high concentrations of sulfate and freeze–thaw conditions, such as salt lake environments and heavily saline soil areas.

Magnesium phosphate cement (MPC) is an inorganic cementitious material with phosphate as a bonding phase. It is produced by dead-burned magnesium oxide (an alkali component), soluble phosphate (an acid component), and additives in a certain proportion through an acid–base chemical reaction under acidic conditions [3]. Compared with Portland cement, MPC has advantages such as fast hardening, high early strength, strong adaptability to ambient temperatures, small volume deformation, performance equaling Portland cement-based materials, wear resistance, frost resistance, and reinforcement protection [4,5,6,7,8]. It has been popularized and applied to repair and reinforce concrete structures [5,7]. As a concrete structure repair material, its durability in a service environment should be considered. Experimental research on the frost resistance and salt corrosion of MPC systems has been undertaken [8,9,10,11,12,13]. Yang et al. [8] soaked one side of an MPC-based specimen in a 3% sodium chloride solution and subjected it to freeze–thaw cycles of freezing at (−20 ± 2) °C for 3 h and thawing at (20 ± 5) °C for 3 h to evaluate its salt freeze–thaw erosion resistance. It was found that MPC-based materials have higher resistance to salt-freezing erosion than ordinary Portland cement concrete without air-entraining. Ding et al. [10] put an MPC mortar sample and an ordinary Portland cement mortar sample into a calcium chloride solution with a concentration of 4%. After the samples were frozen for 16 h and thawed for 8 h, it was found that after 30 freeze–thaw cycles, the surface of the Portland cement sample was seriously denuded, whereas the surface of the MPC sample was flat. After microstructure analysis, it was inferred that the MPC mortar had good salt freeze–thaw erosion resistance because its water saturation was low, and the pores in its hardened slurry were very small with uniformly distributed closed holes. When frozen, the slurry provided buffer space for the squeezed water and reduced the expansion pressure inside the matrix. Li et al. [11] studied the strength development and microstructure evolution process of MPC paste and mortar in water, a NaCl solution, and a Na_2_SO_4_ solution. They found that the influence of the solutions on strength decreased as follows: water > NaCl solution > Na_2_SO_4_ solution. Adding quartz sand reduced the compactness and strength of the MPC microstructure, whereas fly ash had a positive effect on those features. The project team [12,13,14] also studied the strength and volume deformation of MPC paste samples immersed in freshwater, seawater, and a 5% Na_2_SO_4_ solution for a long time. They found that the MPC paste had good resistance to water, sea water, and sulfate, and fly ash could further improve the paste’s corrosion resistance. However, concerning the salt frost resistance of MPC, NaCl solutions have been used as heat transfer media in previous studies, which indicates that MPC has good resistance to sodium chloride salt frost erosion [8,10]. In typical use environments such as an ocean, a salt lake, and groundwater, in addition to chlorine salt, sulfate, especially Na_2_SO_4_, also exists universally. In the early stage of the present study, the project team studied the strength development, volume deformation, and mass loss of MPC slurry subjected to a freeze–thaw cycle of water, 3.5% NaCl, and 5% Na_2_SO_4_ solution. The MPC paste specimen’s performance in the 5% Na_2_SO_4_ solution deteriorated the most, but adding an appropriate amount of limestone powder and silica fume significantly improved the sulfate freeze–thaw resistance of the paste. In previous research, potassium magnesium phosphate cement (MKPC) [15] has been prepared with a controllable setting time and a two-stage hydration-heat release. That provided a premise for applying and expanding MKPC-based materials. Unfortunately, considering that most MPC-based materials used for concrete structure repair are MPC mortar or MPC concrete containing aggregate, until now, no research has been done on the frost resistance and sulfate freeze–thaw resistance of MPC paste containing aggregate. Therefore, in this study MKPC mortar specimens and Portland cement mortar specimens of the same strength grade were prepared with river sand. The sulfate freeze–thaw resistance of MKPC mortar was evaluated by comparing the physical and mechanical properties of the two mortar specimens after sulfate freeze–thaw action.

## 2. Materials and Methods

### 2.1. Raw Materials

The dead-burned magnesia used in this study was obtained from the magnesia plant at the Huanren Dongfanghong hydropower station in Dalian City, Liaoning Province, China. The magnesia was ground in a ball mill for 30 min, and its specific surface area was 230 m^2^·kg^−1^. The oxide composition was analyzed by X-ray fluorescence and is shown in Table 1.

The industrial-grade potassium dihydrogen phosphate used was provided by Lianyungang Geli Chemical Co., Ltd. (Lianyungang, China), and was a colorless or white columnar crystal with a main particle size range of 40/350 to 60/245 (mesh/μm). The composite retarder was made in the study laboratory [14] and comprised borax, disodium hydrogen phosphate dodecahydrate, and inorganic chloride salts, all of which were industrial grade and provided by Liaoning Kuandian chemical plant in Dandong, China. The Portland cement was Conch brand 425 ordinary Portland cement produced in Nantong, Jiangsu, China. The fine aggregate used was ordinary river sand with a fineness modulus of 2.53, a particle grading in zone II (JGJ52-2006), a surface density of 2589 kg/m^3^, a bulk density of 1450 kg/m^3^, and a mud content of 0.8%. The water used in the experiment was Yancheng-area tap water, and the sodium sulfate in the corrosion solution was analytical grade.

### 2.2. Preparation of the Mortar

MKPC was prepared by mixing the alkali component, dead-burned MgO powder, and the acid component, potassium dihydrogen phosphate (KDP) (mass ratio: 1.5:1), with a composite retarder that accounts for 13% of the mass of the MgO powder. Each component was calculated and weighed according to the mixing ratio in Table 2, and subsequently stirred with a cement mortar mixer to obtain freshly mixed MKPC mortar and P.O mortar. The fluidity of cement mortar was tested with reference to the GB/T 2419-2005 ‘Cement mortar fluidity test method’. The fluidity of the slurry was measured by the instrument, and the fluidities of the two mortars were both 150 mm. The fresh mortar was poured into various molds (40 mm × 40 mm × 160 mm to test the strength, Φ 50 mm × 150 mm to test water absorption, and 25 mm × 25 mm × 280 mm to test the deformation). The excess slurry was scraped off after vibrating the sample, which was then sealed with plastic wrap and placed indoors for a certain period (MKPC mortar for 5 h; P.O mortar for 24 h); subsequently, the mold was removed. Afterwards, the salt corrosion test was carried out after curing for 28 d at 20 ± 5 °C and a humidity of 60 ± 5%. The 3 and 28 d strengths of the prepared mortar specimens are shown in Table 2.

### 2.3. Experimental Method

Referring to the Standard Test Method for Resistance of Concrete to Rapid Freeze and Thawing (ASTM C666-15), the freeze–thaw test of the MKPC mortar was completed with an HDK-5 concrete freeze–thaw test machine produced by Beijing Naiheng. Existing research on the frost resistance of concrete [16,17] has found that the mass loss rate, strength loss rate (including flexural strength, compressive strength, and splitting tensile strength), and dynamic elastic modulus loss rate of concrete can be used as an evaluation index of deterioration or damage. However, taking the loss rate of flexural strength as the evaluation index of concrete freeze–thaw durability can be timelier and more accurately reflect freeze–thaw-tested concrete’s deterioration degree. Therefore, in this study, 40 mm × 40 mm × 160 mm prism specimens were used for frost resistance tests to evaluate the flexural strength and compressive strength during a freeze–thaw process. Before the test, a group of 40 mm × 40 mm × 160 mm prismatic MKPC mortar specimens (flexural strength greater than 10.0 MPa and compressive strength greater than 60.0 MPa) was poured. During pouring, a hole was reserved along the central axis of the longest side of the specimens. After a specimen was demolded, a temperature sensor was inserted into the reserved hole and sealed with MPC paste. That was the central temperature control standard sample of the specimen during freeze–thaw testing. Water and 5% Na_2_SO_4_ were selected, respectively. Four days before the test, the MKPC test piece (including the standard sample of temperature control in the center of the test piece) was soaked in the corresponding solution until fully saturated. The embedded temperature sensor was connected to the automatic control system of the freeze–thaw test machine. The maximum and minimum temperatures of the test piece center were 10 and −15 °C, respectively. After repeated debugging, a freeze–thaw system of 3.5 and 2.5 h, respectively, was used. The solution was changed in the sleeve every 25 cycles to ensure a stable solution concentration. When the freeze–thaw cycles reached the set number, the test piece’s quality, deformation, and flexural strength were tested, and photographs were taken regularly to record changes in its surface state.

Referring to ASTM C348-2008 ‘Standard Test Method for Flexural Strength of Hydraulic-Cement Mortars’ and ASTM C 349-2008 ‘Standard Test Method for Compressive Strength of Hydraulic-Cement Mortars (Using Portions of Prisms Broken in Flexure)’, the WED-300 electronic universal testing machine was used to test the flexural and compressive strength of MKPC specimens (40 mm × 40 mm× 160 mm). The flexural and compressive loading speeds were controlled in the range of 0.04–0.06 and 2.2–2.6 kN/s, respectively. The flexural (compressive) strength ($R_{fn}$ ($R_{cn}$)) of the MKPC specimen after being subjected to n freeze–thaw cycles in a water and salt solution and the initial flexural (compressive) strength of the MKPC specimen (the saturated surface dryness immersed in the same solution medium compared with the intensity, $R_{f0}$ ($R_{c0}$)) could be obtained, and the residual ratio of the intensity could be found as follows: $K_{f}=\frac{R_{fn}}{R_{f0}}$, $K_{c}=\frac{R_{cn}}{R_{c0}}$.

Referring to the ‘Standard Test Method for Measuring Water Absorption of Hydraulic Cement Concrete’ (ASTM C1585-2013), an electronic balance with an accuracy of 0.01 g was used to weigh the MKPC specimen (Φ 50 mm × 150 mm) after n freezing and thawing cycles in water and salt solution of the saturated surface dry mass ($W_{n}$); then the test piece was placed in a vacuum drying box, dried at 60 °C for 48 h, and cooled naturally. Subsequently, the dry mass ($W_{n}^{*}$) of the test piece was weighed, and the water absorption of the MKPC specimens was as follows:

(1) $$\rho_{n}=\frac{W_{n}-W_{n}^{*}}{W_{n}^{*}}\times 100\%$$

Before the freezing and thawing of the MKPC specimens, a group of MKPC specimens (40 mm× 40 mm × 160 mm, which were marked and used as special test specimens for testing quality) saturated with the immersion solution were taken and weighed using a balance with an accuracy of 0.01 g. The initial saturated surface dry mass ($W_{0}$) and the saturated surface dry mass ($W_{n}$) of the specimen were weighed after n freeze–thaw cycles, and the mass loss of the MKPC after n freeze–thaw cycles was

(2) $$\Delta W_{n}=\frac{W_{0}-W_{n}}{W_{0}}\times 100\%$$

The MKPC samples used in the microscopic analysis were all obtained from the broken test pieces after the strength test. The sediments adhering to the surface of the test pieces were washed with clean water and immersed in absolute ethanol to stop hydration, followed by drying in a vacuum oven at 60 °C. The samples with skin were ground and passed through a 200-mesh sieve, and the phase composition of the MKPC samples was determined using a Japanese Rigaku D/max-RB X-ray diffraction (XRD) instrument. The instrument is produced by Rigaku Corporation, which is located in Tokyo, Japan.The morphology and element distribution of the MKPC hydration products were observed using a QUANTA200 environmental scanning electron microscope produced by FEI Co., Hillsboro, OR, USA. The TG analysis results were obtained using a NETZSCH STA 409 PC/PG type thermal analyzer. In the experiment, nitrogen was used as the protective gas, the heating rate was 10 °C/min from 20 to 700 °C, and α-Al_2_O_3_ was used as the reference. A small piece of the broken specimen was taken after the strength test and soaked in absolute ethanol to stop hydration. The samples were dried in a vacuum oven at 60 °C before preparation for microscopic analysis. The small pieces of the sample with the skin were taken, ground, and passed through a 200-mesh sieve; a D/max-RB X-ray diffractometer was used to determine the phase composition (XRD) of the MKPC sample. The morphology and element distribution of the MKPC hydration products were observed using a QUANTA200 environmental scanning electron microscope (SEM-EDS). The instrument is produced by FEI, USA, and the company is located in Hillsboro, OR, USA.

## 3. Results and Discussion

### 3.1. Strength Development

**Figure 1:**
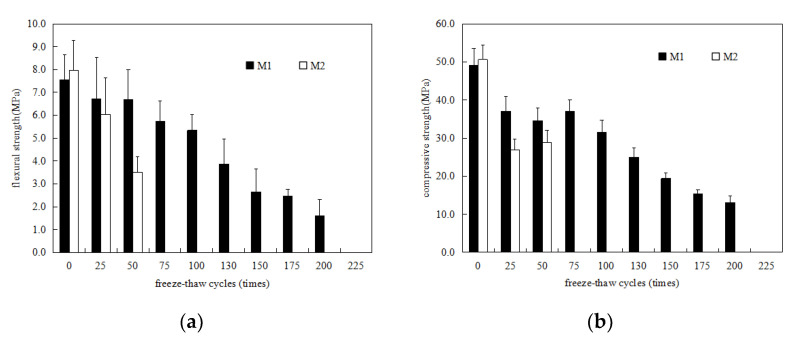
Strength changes in MKPC mortar specimens and P.O mortar specimens in a water-freeze–thaw environment. (**a**) Flexural strength; (**b**) compressive strength.

Figure 1a,b show the flexural and compressive strength changes in MKPC mortar specimens (M1) and P.O mortar specimens (M2) in a water freeze–thaw environment, respectively. It can be seen from Figure 1 that the initial flexural and compressive strengths of M1 are similar to those of M2, indicating that the prepared MKPC mortar specimens and P.O mortar specimens have the same strength level. With increases in the number of freeze–thaw cycles of water, the flexural strength of the M2 specimens decreased rapidly. At 50 freeze–thaw cycles, the flexural strength of M2 was less than 50% of the initial flexural strength, and at 75 freeze–thaw cycles, the M2 specimen was destroyed. The flexural strength of the M1 specimen exhibited a fluctuating downward trend with increases in the number of freeze–thaw cycles. When the number of freeze–thaw cycles reached 75, the flexural strength of the M1 specimen was 75.9% of its initial flexural strength. When the number of melting cycles reached 200, the flexural strength of the M1 specimen was 21.3% of its initial flexural strength, and when the number of cycles reached 225, the M1 specimen was destroyed. Figure 1b shows the compressive strength changes in the M1 and M2 specimens, which are basically consistent with the flexural strength shown in Figure 1a. With increases in the number of freeze–thaw cycles, the compressive strength of M2 decreased rapidly. After 50 freeze–thaw cycles, the compressive strength of M2 was less than 60% of the initial flexural strength. After 75 freeze–thaw cycles, the compressive strength could no longer be measured. The compressive strength of the M1 specimen also exhibited a fluctuating downward trend with increases in the number of freeze–thaw cycles. When the number of freeze–thaw cycles reached 75, the compressive strength of the M1 specimen was 75.1% of its initial flexural strength, and the compressive strength of the specimen was 26.6% of its initial flexural strength; when the number of freeze–thaw cycles reached 225, the M1 specimen was destroyed. The results indicate that the water freeze–thaw resistance of MKPC mortar is significantly higher than that of P.O mortar.

**Figure 2:**
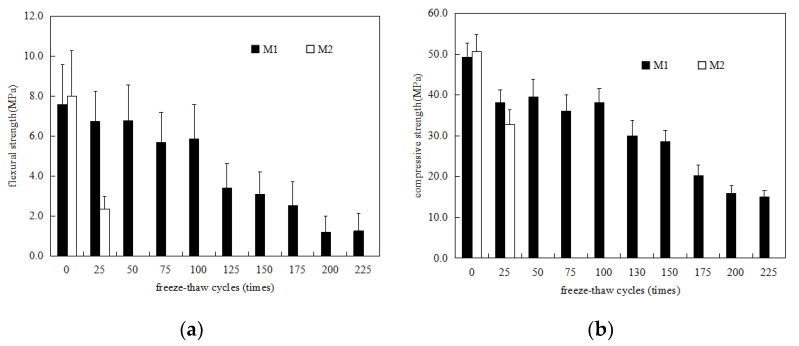
Strength changes in MKPC mortar specimens and P.O mortar specimens in a 5% Na_2_SO_4_ solution freeze–thaw environment. (**a**) Flexural strength; (**b**) compressive strength.

Figure 2a shows the change in the flexural strength of the M1 and M2 specimens in 5% Na_2_SO_4_ solution freeze–thaw environment. It can be seen from the figure that, with increases in the number of freeze–thaw cycles, the flexural strength of M2 decreases rapidly. After 25 freeze–thaw cycles, the flexural strength of M2 was less than 50% of the initial flexural strength. After 50 freeze–thaw cycles, the M2 specimen was destroyed. The flexural strength of the M1 specimen exhibits a fluctuating downward trend with increases in the number of freeze–thaw cycles. When the number of freeze–thaw cycles reached 100, the flexural strength of the M1 specimen reached 75.13% of its initial flexural strength. When the number of melting cycles reached 225, the flexural strength of the M1 specimen was 16.3% of the initial flexural strength. Figure 2b shows the change in the compressive strength of M1 and M2 specimens after different cycles of freezing and thawing in 5% Na_2_SO_4_ solution, which basically reflects the same development law as the flexural strength in Figure 2a. With increases in the number of freeze–thaw cycles, the compressive strength of M2 decreased rapidly. After 25 freeze–thaw cycles, the compressive strength of M2 was less than 75% of the initial compressive strength. Subsequently, the compressive strength could not be measured. When the number of freeze–thaw cycles reached 100, the compressive strength of the M1 specimen reached 77.5% of its initial flexural strength, and when the number of freeze–thaw cycles reached 225, the compressive strength of M1 reached 30.5% of its initial compressive strength. Comparing the results in Figure 2 with the results in Figure 1, the resistance of M1 to sodium sulfate freeze–thaw is better than that to water freeze–thaw, while the resistance of M2 to sodium sulfate freeze–thaw resistance is significantly lower than its water freeze–thaw resistance. The freeze–thaw resistance of MKPC mortar was significantly higher than that of P.O mortar.

When using the rapid freeze–thaw test method, the ice expansion pressure is the main reason for the strength loss in water freeze–thaw mortar specimens [16,17]. C-S-H gel, the main hydration product of Portland cement mortar, is porous; therefore, the open porosity of the hardened body is high. As a result, the freezing pressure increases, resulting in a rapid decrease in the strength of the Portland cement mortar specimen. The main hydration product of MKPC mortar, MKP, has a polycrystalline network lap structure, and the open porosity of its hardened body is significantly lower than that of Portland cement mortar, and environmental water cannot easily penetrate. Under rapid water freezing and thawing conditions, the ice expansion pressure generated in the hardened body is significantly reduced. In a freeze–thaw environment, the MKPC mortar specimen is first subjected to the ice expansion pressure. Since the MKPC mortar specimen is in a water-immersed environment during the thawing process, the MKPC mortar specimen will continue to hydrate the acid–base components and hydrolyze the generated MKP [11,18,19]; because the solubility of MKP is related to temperature, the hydrolysis loss of MKP in a low-temperature water environment will be reduced. The abovementioned effects cause the strength of MKPC mortar to fluctuate, and with increases in the freeze–thaw cycles, the continuous hydration weakens. The water swelling pressure plays a leading role, which results in a decrease in the strength of the MKPC specimen.

The salt freeze–thaw corrosion mechanism of mortar specimens under rapid freeze–thaw conditions is basically the same as that of water freeze–thaw. Salt can reduce the freezing point of water, thereby reducing the ice expansion pressure in the mortar hardened body; however, the hygroscopicity of salt can improve cement-based materials. Internal water saturation, once the supercooled water generated by the salt solution freezes, has a greater destructive force, and the supersaturation of the salt in the pores of the hardened body will also destroy the salt crystals; the aforementioned effects will cause the salt to freeze and thaw Portland cement. Further, the strength loss of the mortar specimen is more serious than that during water freezing and thawing. In a low-temperature sulfate environment, the sulfate radicals that infiltrate into the MKPC mortar hardened body will combine with Mg^2+^ to form hydrated magnesium sulfate crystals and fill the internal pores (see XRD analysis for details). The strength loss of sulfate frozen–thawed MKPC mortar specimens was lower than that of water frozen–thawed MKPC mortar specimens.

### 3.2. Weight Loss

**Figure 3:**
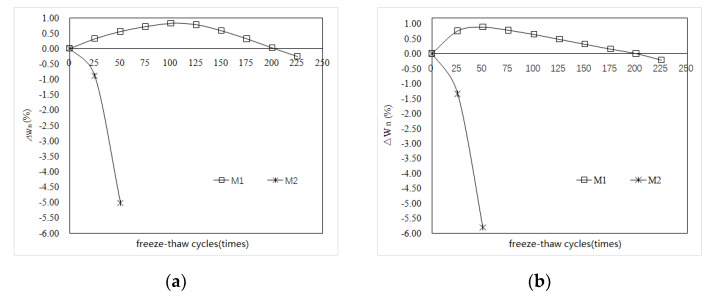
Mass change in MKPC mortar and P.O mortar samples in freeze-thaw environments. (**a**) Water freeze-thaw environment; (**b**) 5% Na_2_SO_4_ freeze-thaw environment.

Figure 3 reflects the changes in the mass of the M1 and M2 specimens with the number of freeze–thaw cycles in water and 5% Na_2_SO_4_ solution. Figure 3a shows that, with increases in the number of freeze–thaw cycles, the mass loss rate of the M2 specimen increases rapidly, and the mass loss exceeds 5% after 50 freeze–thaw cycles. Subsequently, the test was terminated owing to serious damage to the specimen. The mass change rate of the M1 specimen exhibited a trend of first increasing and then gradually decreasing with increases in the number of freeze–thaw cycles. When the water was frozen and thawed 225 times, the mass loss rate of the M1 specimen was 0.26%. It can be seen from Figure 3b that, with increases in the number of freeze–thaw cycles of 5% Na_2_SO_4_ solution, the mass loss rate of the M2 specimen increases faster than that of water freeze–thaw M2, and the mass loss of 50 freeze–thaw cycles also exceeds 5%. The mass change rate of the M1 specimen increased first and then decreased with increases in the number of freeze–thaw cycles. When the freeze–thaw cycles reached 225, the mass loss rate of the M1 specimen was 0.21%; the quality loss was slightly lower. The results show that the mass loss of the P.O mortar specimens is much larger than that of MKPC mortar, and the mass loss of salt freeze–thaw specimens is higher than that of water freeze–thaw specimens; the quality loss exceeds the 5% damage standard. MKPC mortar has superior water and sulfate freeze–thaw resistance; the latter is superior to the former. When the freeze–thaw cycles in water and 5% Na_2_SO_4_ solution are cycled 225 times, the mass loss of MKPC mortar is still much lower than the 5% destruction standard.

**Figure 4:**
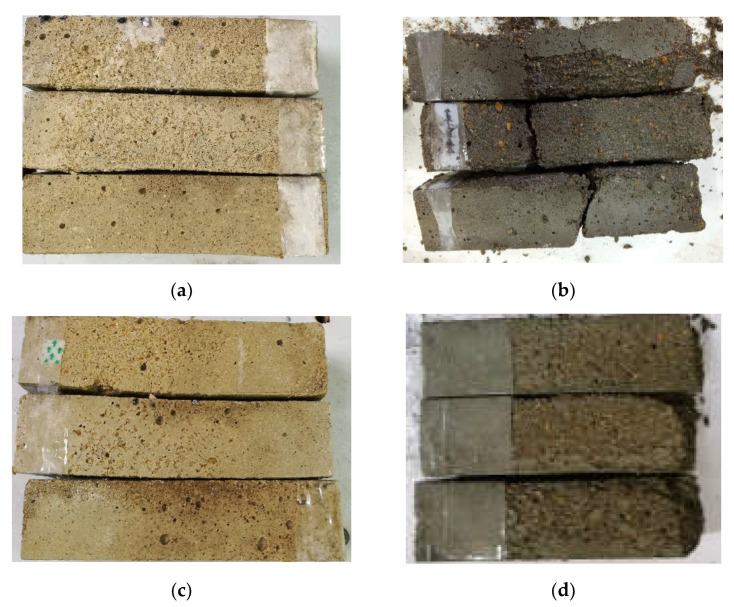
Appearance of MKPC mortar specimens and P.O mortar specimens in a freeze–thaw environment. (**a**) M1, 225 water freeze-thaw cycles; (**b**) M2, 50 water freeze-thaw cycles; (**c**) M1, 225 salt-thaw cycles; (**d**) M2, 25 salt-thaw cycles.

Figure 4 shows the appearance of the M1 and M2 specimens subjected to freeze–thaw cycle tests in water and 5% Na_2_SO_4_ solution until the end of the test. After 225 water freeze–thaw cycles, the epidermis of the M1 specimen was basically peeled off, the exposed surface capillary was large, the specimen had a small number of cracks, and the edges and corners had a small number of defects; after 50 water freeze–thaw cycles, the edges and corners of the M2 specimen were severely damaged. The specimen broke automatically when it was taken out, and its internal structure and crispness can be seen from the exposed surface. The shape of M1 after 225 cycles of sulfate freezing and thawing was basically intact, and the degree of skin peeling was less than that of M1 frozen and thawed by water. After 25 cycles of sulfate freezing and thawing, the edges and corners of the M2 specimen were severely damaged, the epidermis was completely peeled off, the exposed surface had large pores, and the structure was loose.

The mass loss of cement-based materials subjected to freezing and thawing is one of the main evaluation indicators that reflects their freezing resistance. When the number of freeze–thaw cycles is small, the surface damage of the mortar specimen is small. With increases in the number of freeze–thaw cycles, the surface of the specimen begins to peel off gradually, and the quality of the specimen also decreases. After the aggregates were exposed on the surface of the specimen, the micro-cracks further developed to the interior regions, the spalling and damage to the specimen intensified, and the mass loss also increased. The mass loss of Portland cement mortar specimens entirely corresponds to the spalling defects of the specimens. The mass loss of MKPC mortar subjected to freeze–thaw cycles was also attributed to the spalling and angular defects on the surface of the specimen, but due to the dense structure of MKPC mortar, the ice expansion pressure and salt crystallization pressure generated during the freeze–thaw cycles were small, along with the surface erosion and crystallization pressure. Therefore, defects were significantly reduced, and quality loss was also small.

### 3.3. Water Absorption

**Figure 5:**
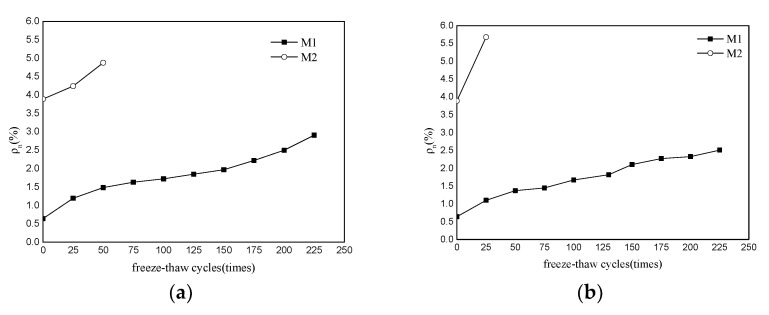
Water absorption changes in MKPC mortar samples and P.O mortar samples under freezing and thawing conditions. (**a**) water freeze-thaw; (**b**) 5% Na_2_SO_4_ freeze-thaw.

Figure 5 shows the changes in the water absorption of the two mortar specimens after different numbers of freeze–thaw cycles. In Figure 5a, the water absorption of the M2 specimen is close to eight times that of the M1 specimen before the freeze–thaw cycling begins. With an increase in the number of water freeze–thaw cycles, the water absorption rate of the M2 specimen increased rapidly, and after 50 water freeze–thaw cycles, the water absorption rate reached 4.88%. The water absorption rate of the M1 specimen increased gradually with increases in the number of water freeze–thaw cycles. After 225 water freeze–thaw cycles, the water absorption rate was 2.91%. It can be seen from Figure 5b that the change trend of water absorption for M1 and M2 specimens after freeze–thaw cycles in a 5% Na_2_SO_4_ solution is basically the same as that after freeze–thaw cycles in water. The water absorption rate of the M2 specimen increased rapidly, and after 25 freeze–thaw cycles, the water absorption rate reached 5.68%, which exceeded the water absorption rate of the specimen after 50 freeze–thaw cycles in water. The water absorption rate of M1 gradually increased with an increase in the number of freeze–thaw cycles in 5% Na_2_SO_4_ solution. After 225 freeze–thaw cycles, its water absorption rate was 2.51%, which was lower than its water absorption rate after 225 freeze–thaw cycles in water. The open porosity of a solid material can reflect the advantages and disadvantages of its pore structure; for the same solid material, the water absorption and open porosity are numerically uniform. The test results in Figure 5 indirectly reflect the change in the open porosity of the specimen. Before the start of the freeze–thaw test, the open porosity of M1 was significantly lower than that of M2, which can be attributed to the low water–cement ratio of MKPC mortar and the polycrystalline ceramic structure of its main hydration products. With increases in the number of freeze–thaw cycles, the ice expansion pressure and salt crystallization pressure lead to the gradual deterioration of the pore structure of the hardened body. The higher the open porosity of the specimen, the more significant the deterioration of the pore structure.

### 3.4. XRD Analysis

**Figure 6:**
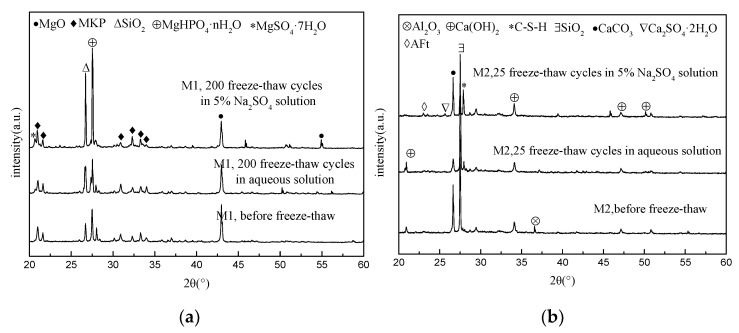
XRD patterns of MKPC mortar samples and P.O mortar samples. (**a**) M1; (**b**) M2.

Figure 6 shows the XRD patterns obtained from the analysis of the hardened body powder of the broken M1 and M2 specimens before and after 200 (25) freeze–thaw cycles in water and a 5% Na_2_SO_4_ solution. Figure 6a shows the XRD analysis results for M1. The types and positions of the main diffraction peaks in the M1 samples subjected to natural curing, 200 water freeze–thaw cycles, and 200 sulfate freeze–thaw cycles are approximately the same, with characteristic peaks of unreacted MgO and sacrificial peaks of the hydration product MgKPO_4_·6H_2_O (MKP), as well as the main characteristic peaks of MgHPO_4_·nH_2_O and SiO_2_. The characteristic peaks of MgSO_4_·7H_2_O could also be seen for the M1 sample after 200 sulfate freeze–thaw cycles, indicating that sulfates had penetrated the matrix and reacted with Mg^2+^. Figure 6b shows the XRD analysis result of M2. In the natural conservation M2 sample, characteristic peaks of CSH, Ca(OH)_2_, CaCO_3_, Al_2_O_3_, and SiO_2_ mainly exist. The characteristic peaks of AFt and CaSO_4_·2H_2_O can also be seen, indicating that the sulfate radicals in the solution are involved in the chemical reaction.

### 3.5. TG Measurements

**Figure 7:**
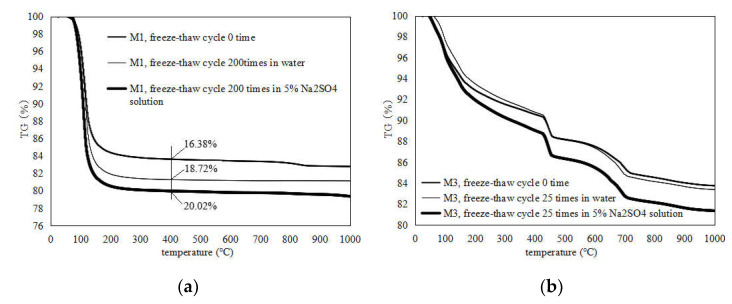
TG of M1 and M2 samples after different numbers of cycles of freeze–thaw in water and 5% Na_2_SO_4_. (**a**) M1; (**b**) M2.

Figure 7 shows the TG curve obtained from the analysis of the powders of the broken specimen before and after 200 (25) freeze–thaw cycles in water and a sulfate solution. Figure 7a shows the TG curve of M1 before the freezing and thawing cycles, 200 iterations of water freezing and thawing, and 200 iterations of 5% Na_2_SO_4_ solution freezing and thawing cycles. There is an obvious mass loss in the three MKPC mortar samples at about 100 °C, which is caused by the loss of crystallization water in MKP crystals [20]. The weight losses of M1 subjected to different environmental conditions at about 100 °C differ significantly. The weight loss (18.72%) of the M1 sample after 200 cycles of water freezing and thawing is significantly higher than that of the M1 sample before freezing and thawing (16.38%), indicating that MKPC mortar still experiences the continuous hydration of acid–base components in a freezing and thawing environment, along with hydrolysis losses of MKP [21]. However, owing to the decrease in MKP solubility in water at low temperatures, the continuous hydration of incomplete acid–base reaction components is still dominant, resulting in the increase in MKP content in the MKPC mortar sample. The weight loss (20.02%) of the M1 sample after 200 freeze–thaw cycles in sodium sulfate solution is significantly higher than that of the M1 sample with 200 water freeze–thaw cycles (18.72%), indicating that the MKP content is further improved. The pH of the sodium sulfate solution slightly increases in low-temperature environments, which makes the newly formed MKP in the MKPC mortar easier to crystallize, reducing the dissolution of the generated MKP [22,23]. Figure 7b shows the TG curve of M2 after freezing and thawing cycles: 25 water freeze–thaw cycles and 25 Na_2_SO_4_ freeze–thaw cycles. In comparison to MKPC mortar, the TG curves of the P.O mortar follow a completely different law. The weight loss of the P.O mortar at about 80 °C is caused by water loss of the C-S-H gel, and the weight loss at 400–500 °C is caused by water loss of Ca(OH)_2_. The TG curve of the M2 samples after 25 water freezing and thawing cycles is basically consistent with that of the M2 samples before freezing and thawing, indicating that the freezing and thawing process has little effect on the composition of the hardened body, i.e., the failure process is mainly a physical action. The TG curve of the M2 frozen and thawed in sulfates 25 times increases slightly at about 80 °C, which could be due to the decrease in the freezing point of the salt solution and the continuous hydration in the low-temperature salt solution.

### 3.6. SEM-EDS

**Figure 8:**
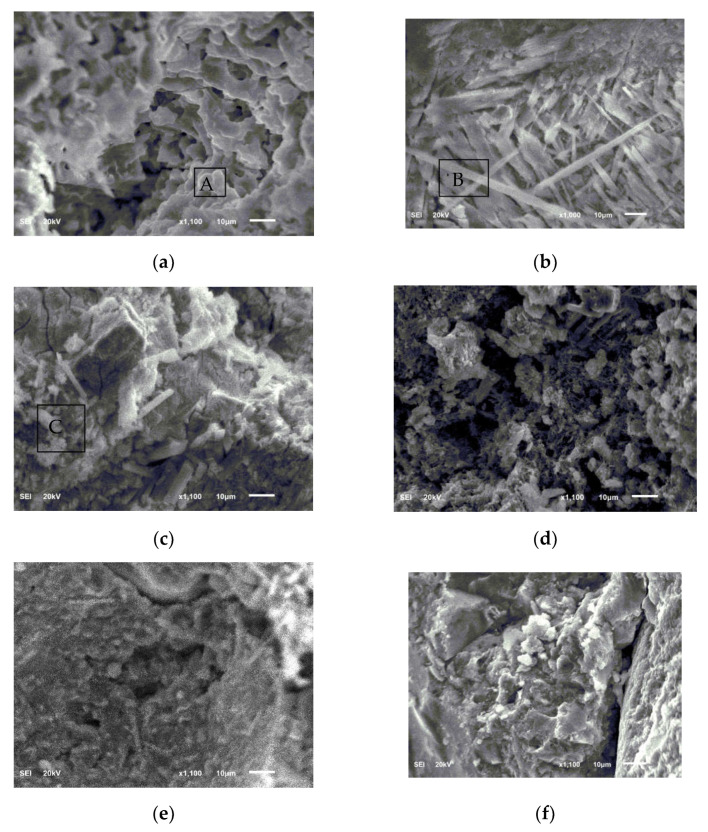
SEM images of MKPC mortar samples and P.O mortar samples. (**a**) M1, before freeze–thaw; (**b**) M1, after 200 freeze–thaw cycles in water solution; (**c**) M1, after 200 freeze–thaw cycles in 5% Na_2_SO_4_ solution; (**d**) M2, before freeze–thaw cycles; (**e**) M2, after 50 freeze–thaw cycles in water solution; (**f**) M2, after 50 freeze–thaw cycles in 5% Na_2_SO_4_ solution. A–C are EDS analysis of corresponding areas, see Table 3 for details.

Figure 8 shows the SEM image obtained by taking small samples of broken M1 and M2 specimens after being subjected to different environmental conditions. Figure 8a shows the SEM image of the fracture surface of the M1 sample before freezing and thawing; it can be seen that the hydration products in the fractured pores are less filled and the structure is loose, with many holes in the area. The P and K elements have a similar molar ratio, and O and Mg content are relatively high (Table 3). The XRD results show that the main hydration products of the MKPC slurry are MKP and unreacted MgO. Figure 8b shows an SEM image of M1 frozen and thawed in water 200 times; it can be seen that the hydration products in the cross-section pores are filled with a high amount of mainly short-range ordered needle-like crystals. The degree of crystallization is significantly higher than that in Figure 8a, indicating that there is still a continuous hydration reaction during the freeze–thaw process. Region B is mainly composed of O, Mg, P, and K elements, where the molar ratio of Mg, P, and K is similar (Table 3), which reflects the main hydration product of MKPC slurry, MKP. There are many cracks on the stomatal wall of the sample section, which could be caused by the compressive stress of ice expansion. Figure 8c shows an SEM image of M1 frozen–thawed in 5% Na_2_SO_4_ solution 200 times; it can be seen that the filling amount of hydration products in the fractured pores is high, which mainly comprise columnar crystals and amorphous phases, and the filling degree is obviously high. In Figure 8a, it can be seen that there is still a continuous hydration reaction during the salt freeze–thaw process. Region C is composed of O, Mg, Na, Cl, P, K, and S elements, which reflects the main hydration product, MKP, where Na and Cl arise from composite retarders, and the presence of S confirms that sulfate radicals are mixed in the water chemical products (Table 3). Figure 8d shows an SEM image of the fracture surface of the M2 sample before freezing and thawing. It can be seen from the figure that the hydration products in the fractured pores are composed of fine crystals and amorphous phases. There are obvious differences in appearance, and there are more pores in the hydration products. Figure 8e shows an SEM image of the M2 sample frozen and thawed 50 times in clean water. There are obvious cracks on the pore wall, which could be caused by ice expansion stress. Figure 8f is the SEM image of M2 frozen and thawed in 5% Na_2_SO_4_ solution 50 times. The crack texture on the pore wall of the cross-section was more severe.

## 4. Conclusions

(1) The P.O mortar specimen (M2) completely lost its strength after 75 freeze–thaw cycles in water and 50 freeze–thaw cycles in 5% Na_2_SO_4_ solution. The MKPC mortar specimen (M1) with the same strength grade was frozen and thawed in water 75 times and 5% Na_2_SO_4_ solution 100 times, where the residual strength rate was higher than 75%. After 50 freeze–thaw cycles in water and 5% Na_2_SO_4_ solution, the mass loss of the M2 specimens exceeded the 5% damage standard, while the mass loss of M1 was much lower than 5%. These results show that the water freeze–thaw resistance and sulfate solution freeze–thaw resistance of MKPC mortar specimens is much higher than that of Portland cement mortar specimens, and the salt freeze–thaw resistance is better than the water freeze–thaw resistance.
(2) Before the freeze–thaw test, the open porosity of the MKPC mortar hardened body was significantly lower than that of the Portland cement mortar, and environmental water could not easily infiltrate. In a supercooled water environment, there will still be incompletely reacted acid–base components in the MKPC hardened body to continue the hydration process, and the hydrolysis loss of the generated MKP in the low-temperature water environment will be alleviated, and the newly generated MKP will fill the pores of the hardened body. The MKPC hardened body tends to be dense; the aforementioned effects make the MKPC mortar’s anti-water freeze–thaw and sulfate freeze–thaw properties significantly better than those of P.O mortar.

## Figures and Tables

**Table 1:** Oxide composition of dead-burned magnesia powders.

Oxide Composition	MgO	SiO_2_	CaO	Fe_2_O_3_	Al_2_O_3_	Na_2_O	TiO_2_	Others
Content/%	91.85	3.68	3.14	0.865	0.17	-	-	0.285

**Table 2:** Mixing ratio of MKPC mortar.

Code	P.O 42.5	MKPC	WS/ Wcement	WW/ Wcement	Flexural Strength/MPa		Compressive Strength/MPa	
					3 d	28 d	3 d	28 d
M1	-	1.00	1.50	0.20	6.16	7.96	35.1	51.8
M2	1.00	-	3.0	0.5	6.03	8.15	30.7	53.3

Note: S denotes sand, and W denotes water.

**Table 3:** Micro-area element distribution of MKPC mortar samples.

Element	Area	O	Mg	S	P	Cl	K	Na
Atomic %	A	77.73	9.74	-	6.65	-	5.88	-
	B	70.21	11.93	-	8.78	-	9.08	-
	C	69.37	7.22	1.45	9.69	0.27	9.60	2.4

## Data Availability

Data are contained within the article.
